# Numerical simulation of embryo transfer: how the viscosity of transferred medium affects the transport of embryos

**DOI:** 10.1186/s12976-018-0092-y

**Published:** 2018-10-05

**Authors:** Dali Ding, Weiping Shi, Yang Shi

**Affiliations:** 1 College of Mathematics, Jilin University, Changchun, 130012 People’s Republic of China; 2 Division of Biostatistics and Data Science, Department of Population Health Sciences, Medical College of Georgia, Augusta University, Augusta, GA 30912 USA

**Keywords:** Embryo transfer, Viscosity of transferred medium, Multiphase flow, Mixing flow, Dispersion pattern, Numerical simulation, In vitro fertilization

## Abstract

**Background:**

Embryo transfer (ET) is a key step of assisted reproductive procedures, where the transferred medium containing the embryos is injected into the uterine cavity through a transcervical catheter and blended with intrauterine fluid in the uterine cavity. This procedure determines the delivery sites of embryos in the uterine cavity and has crucial impact on the implantation. Due to practical restrictions and ethical issues, it is often difficult to perform an in vivo study in humans to examine factors that affect the motions and delivery of embryos during ET. Alternatively, mathematical modeling is a powerful tool to that end.

**Results:**

A computational model is developed to simulate the intrauterine mixing flow and track the embryo motions. Two important factors affecting the intrauterine flow are studied via this model: the viscosity of the transferred medium and the injection speed. Numerical results show that the dispersion pattern and the final delivery sites of the embryos are significantly influenced by the viscosity of the transferred medium. Specially, increasing the transferred medium viscosity close to that of the uterine fluid can enhance the probability that the embryos are delivered close to the fundus and keep them from being dragged backward to the cervix during catheter withdrawal. In addition, a slow injection speed can lower the driving force on the embryo during ET, which can prevent the embryo from being injured.

**Conclusions:**

Based on our study, the practice of using a transferred medium with similar viscosity to that of the uterine fluid and a slow injection speed is recommended for real embryo transfer procedures in clinic.

## Background

Embryo transfer (ET) is the last step of assisted reproduction procedures. During ET, a catheter is loaded with the transfer medium containing 1–5 embryos, inserted into the uterine cavity via the cervical canal, and then injected its loading and withdrawn from the uterine cavity immediately. Despite the successful in vitro fertilization (IVF) rate of greater than 80% in laboratory, the pregnancy rate per ET is unfortunately as low as 44% [1]. To increase the chances of pregnancy after ET, investigators are making efforts to improve the operational techniques nowadays.

Several factors have a significant influence on the pregnancy rate of ET, such as the catheter placement, the loading of the catheter, the speed of injection, the operation of catheter withdrawal and the uterine contraction [2–4]. Due to the non-self-propel feature of the embryo, its transport depends on the intrauterine flow driven by the injection of the catheter load. To keep the embryo from falling through the cervix into vagina or entering the fallopian tubes, several procedures for controlling the operational details of the injection are suggested, which include setting the distance between the catheter tip and the fundus between 15 to 20 mm, and controlling the transferred volume below 30 μL [2, 5–7]. In addition, the content of the intrauterine fluid is another major factor that affects the transport of the embryos. The injection-driven flow in the uterine cavity is a multiphase flow of the uterine fluid and the transferred medium. The uterine fluid is a gel-like fluid with a relative high viscosity about 1 Pa·s due to high content of glycosaminoglycan [8, 9], while the viscosity of the widely used transferred medium, the normal saline, is about 0.001 Pa·s [1], which is much lower than that of the uterine fluid. The viscosity of the transferred medium can be increased by adding hyaluronan, a major type of glycosaminoglycan in follicular, oviductal and uterine fluids [10]. Several studies tried to adjust the viscosity of the transferred medium in order to increase the implantation rate. A clinical study by Menezo et al. reported that increasing the viscosity of the medium to 120 times that of water did not appear to increase the success rate of ET [11], whereas another study by Eytan et al. observed that dispersion of the embryos during ET towards the cervix was efficiently avoided by increasing the medium viscosity similar to that of uterine fluid [12].

Because of practical restrictions and ethical issues, it is often difficult to perform an in vivo study in humans to monitor the motions and delivery sites of embryos during ET. Furthermore, even reported in vivo studies sometimes show contradictory results as mentioned above. Alternatively, mathematical modeling is a powerful tool for simulating and investigating the kinetic features of embryos in ET, and several models have been developed in the last decade. Yaniv et al. proposed a computational model to simulate ET within the uterine cavity and investigated the motions of embryos, where a two dimensional channel with oscillating walls was employed to represent the uterine peristalsis [13]. Furthermore in their subsequent study [14], the intrauterine fluid flow patterns in transporting the embryos to the implantation sites were studied through numerical simulations. Their results revealed that the embryo transport patterns were strongly affected by the closed fundal end [14]. In another study, the ET procedure was simulated based on a two dimensional computational model, and numerical results showed that the embryo transport were strongly affected by the procedural parameters during ET [15].

Though the above models considered many important parameters during the ET procedures, an obvious drawback of them is that the transferred medium was assumed to have the same properties as the uterine fluid, which is often not true in real practice. Therefore, it is necessary to study the influence of the transferred medium. In this paper, a computational model for simulating the intrauterine mixing flow is developed to evaluate how the viscosity of the transferred medium affects the transport of embryos during ET. In particular, a homogeneous multiphase model is adopted in order to simulate the mixing flow in uterine cavity and a particle tracking technique is employed to predict the trajectories of the embryos. In the next section, the model and the numerical methods used are presented in detail, followed by the simulation results and discussion. Conclusion and recommendation of the optimal condition for real ET procedures considering the parameters studied in this work are given at the end.

## Methods

### The geometric model

**Fig. 1:**
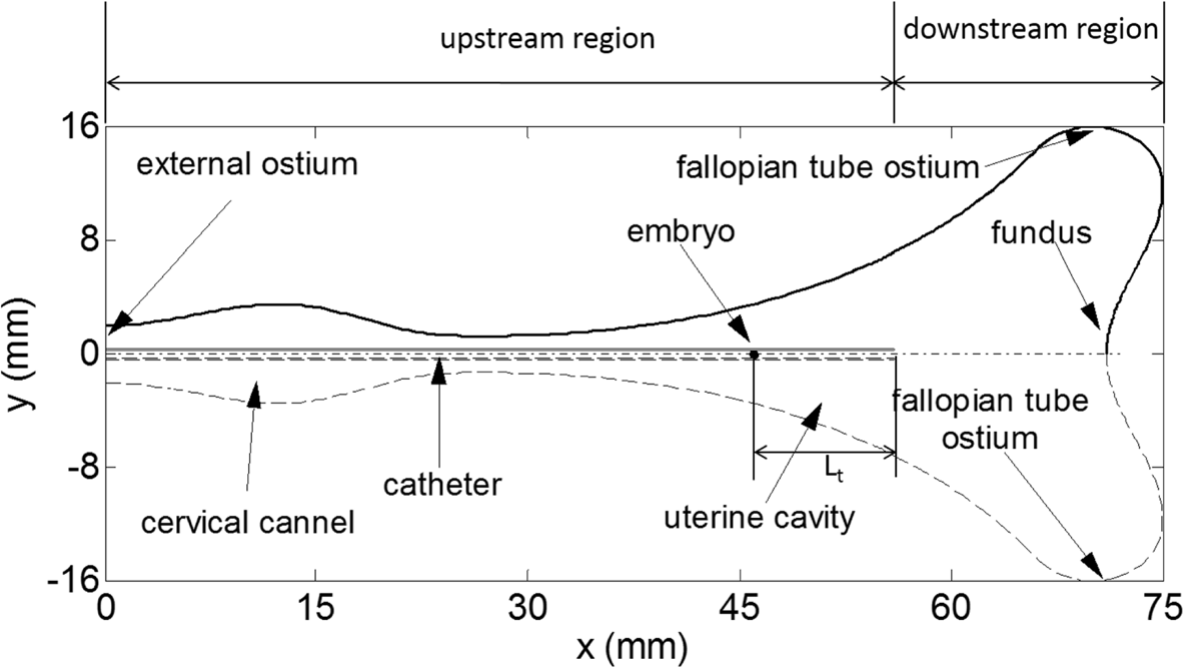
Geometric model used in the simulations: The schematic represents the cross-section of an average-sized uterine cavity with a catheter placed along the midline

A two-dimensional region is established to represent the mid-sagittal cross-section of an average sized uterine cavity, which is shown in Fig. 1. The geometric parameters of this region are set according to [15]. Specifically, the shape of the uterine cavity is approximated by a triangle with a base of 32 mm and a height of 50 mm, which is connected with the outside of the body via the cervical channel of 25 mm in length. There are two openings of 0.3 mm on the boundary of uterine cavity representing the fallopian tube ostia and another opening of 4 mm at the left end of the cervical channel representing the external ostium of the uterus. A catheter with internal and external diameters of 0.6 and 0.8 mm, respectively, is placed along the midline of the uterine cavity (denoted by the dashed line in Fig. 1). The length of the catheter inside the uterine cavity is set as 56 mm and the distance between the catheter tip and the uterine fundus is 15 mm (Fig. 1). Based on the fact that both the shape of the uterine cavity and the flow inside are symmetric about the midline [12, 15], only half of the uterine cavity, i.e. the zone above the midline in Fig. 1, is chosen as the computational domain in this study.

### The computational model

During the ET procedures, both the transferred medium and uterine fluid can be considered as incompressible fluid in the intrauterine environment. The intrauterine mixing flow is expected to have a Reynolds number in the order of 10 [15] and thus is regarded as incompressible laminar flow. Therefore, the mass and momentum conservation equations for the intrauterine mixing flow can be written as

1 $$ \frac{\partial }{\partial t}\left({\rho}_m\right)+\nabla \cdot \left({\rho}_m{\overset{\rightharpoonup }{u}}_m\right)=0, $$

2 $$ \frac{\partial }{\partial t}\left({\rho}_m{\overset{\rightharpoonup }{u}}_m\right)+\nabla \cdot \left({\rho}_m{\overset{\rightharpoonup }{u}}_m{\overset{\rightharpoonup }{u}}_m\right)=-\nabla p+\nabla \cdot \left({\mu}_m\nabla \left({\overset{\rightharpoonup }{u}}_m+{\overset{\rightharpoonup }{u}}_m^T\right)\right), $$

where *p* is the pressure, *ρ*_*m*_, *μ*_*m*_ and ${\overset{\rightharpoonup }{u}}_m$ are the density, viscosity and velocity vectors of the mixture fluid, respectively. The volume fraction of the transferred medium, denoted by *α*, is calculated in the computational domain and used to distinguish the transferred medium and uterine fluid in a control volume. Since the time of embryo injection is very short during ET, the intrauterine flow is dominated by convective flow, whereas diffusion between the transferred medium and the uterine fluid is negligible and thus is ignored in this work. Because of the miscibility of the transferred medium and the uterine fluid, it is reasonable to assume that the velocities of the two species of fluids are equal locally. Therefore, the volume fraction equation of transferred medium can be expressed as

3 $$ \frac{\partial }{\partial t}\left({\alpha \rho}_1\right)+\nabla \cdot \left({\alpha \rho}_1{\overset{\rightharpoonup }{u}}_m\right)=0, $$

where *α* and *ρ*_1_ are the volume fraction and density of the transferred medium, respectively. To close Eqs. (1)–(3), *ρ*_*m*_ and *μ*_*m*_ can be obtained from

4 $$ {\rho}_m={\alpha \rho}_1+\left(1-\alpha \right){\rho}_2, $$

5 $$ {\mu}_m={\alpha \mu}_1+\left(1-\alpha \right){\mu}_2. $$

where the subscript 1 and 2 denote the transferred medium and uterine fluid, respectively. Equations (1) to (5) constitute the governing equations of intrauterine mixing flow. This model is consistent with the homogeneous multiphase model, which is the mixture model ignoring velocity difference between phases [16]. Therefore, commercial software packages for the homogeneous multiphase model can be conveniently used for simulating the flows based on this model.

### Boundary conditions

All the walls are assumed the no-slip and no-penetration condition. Specially, moving wall conditions are defined on the tip, inner and outer walls of the catheter during the process of catheter withdrawal. The openings that represent the fallopian tube ostium and external ostium are chosen as the outflow condition with a constant pressure. The left opening of the catheter is specified as the inflow condition with given inlet velocity.

### Prediction of embryo trajectories

An embryo is considered as an inertial particle with a spherical shape. Due to the small size of the embryo, its effect on the intrauterine flow is ignored in the numerical simulations. The dynamic equation of the embryo that represents the equality of the drag force and inertial force on the embryo is written as

6 $$ \frac{d{\overset{\rightharpoonup }{v}}_e}{dt}=\frac{3{\mu}_m{C}_D{\operatorname{Re}}_r}{8{\rho}_e{r}_e^2}\left({\overset{\rightharpoonup }{u}}_m-{\overset{\rightharpoonup }{v}}_e\right), $$

where ${\overset{\rightharpoonup }{v}}_e$, *ρ*_*e*_, *r*_*e*_ are the velocity vector, density and radius of the embryo, respectively, *C*_*D*_ is the drag coefficient of the embryo and Re_*r*_ is the relative Reynolds number defined as

7 $$ {\operatorname{Re}}_r=\frac{2{\rho}_m{r}_e\mid {\overset{\rightharpoonup }{v}}_e-{\overset{\rightharpoonup }{u}}_m\mid }{\mu_m}, $$

In addition, the drag coefficient *C*_*D*_ is a function of Re_*r*_ and its formula can be found in the work of Morsi and Alexander [17]. Moreover, the trajectory of the embryo is predicted by

8 $$ \frac{d{\overset{\rightharpoonup }{x}}_e}{dt}={\overset{\rightharpoonup }{v}}_e, $$

where ${\overset{\rightharpoonup }{x}}_e$ is the displacement vector of the embryo. Given the initial position, the mass and radius of an embryo, we can calculate its trajectory by the numerical temporal integration of Eqs. (6) and (8).

**Fig. 2:**
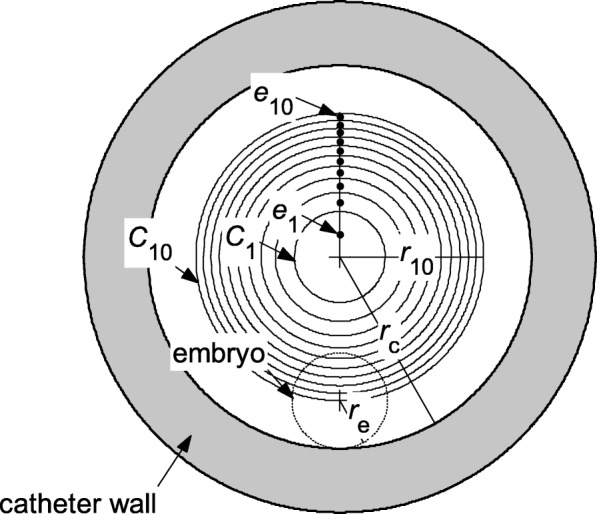
Illustration of the initial positions of the embryos in the catheter in the simulations: The small dashed-line circle represents the shape of an embryo. The 10 black dots (*e*_1_, …, *e*_10_) represent the centers of the 10 embryos used in the simulations. The shaded annulus represents the catheter wall

The embryo’s initial distance to the catheter axis at the start of injection is a parameter that can influence the trajectory of the embryo during ET substantially [14, 15], which needs to be specified in our simulations. However, the initial distance of an embryo to the axis of catheter is uncertain in a real ET. To take this randomness into account, we set the embryo’s initial position in the following way. As shown in Fig. 2, a cross section of the catheter, 10 equal-area zones (one circle at the center and nine annuluses around it) are divided and the initial position of the embryo (represented by the small dashed-line circle in Fig. 2) is assumed to appear in each of 10 zones with equal probability. To ensure that the area of each zone is equal, the radius *r*_*i*_ of the *i*th circle *C*_*i*_, (*i* = 1, 2, …, 10, Fig. 2) is determined as

9 $$ {r}_i={i}^{0.5}{10}^{-0.5}\left({r}_c-{r}_e\right),i=1,2,\dots, 10 $$

where *r*_*c*_ is the interior radius of the catheter. In our simulations, we track the trajectories of 10 embryos denoted as *e*_1_, …, *e*_10_, and we assume the centers of the 10 embryos are located initially at the midpoints of the 10 segments divided by the circles *C*_1_–*C*_9_ along the radius, where *e*_1_ is the closest one to the catheter axis, *e*_10_ is the farthest one, and other embryos are between *e*_1_ and *e*_10_ (Fig. 2). Here we calculate the trajectory of one embryo in each numerical simulation, and we calculate the numerical temporal integration of Eqs. (6) and (8) 10 times in order to obtain the trajectories of those 10 different embryos separately.

### Numerical methods

The nonlinear governing equations (Eqs. (1)–(3)) are discretized in space and time and solved using the finite volume software package FLUENT. The first order upwind and the first order implicit schemes are adopted to discretize the convection and unsteady terms, respectively, and the pressure term in momentum conservation equation is handled by the Semi-Implicit Method for Pressure-Link Equations (SIMPLE) algorithm. A double precision solver is applied to improve the accuracy of the simulations. By using GAMBIT, the program used to build geometry model and generate mesh, the geometric region of uterine cavity (Fig. 1) is discretized into structured girds of 19,280 quadrangular cells, and the grids in the vicinity of the catheter tip are refined. The displacement of the embryo is computed by trapezoidal integration of Eqs. (6) and (8) in each time step of 0.0001 seconds (abbreviated as “s” below). The embryo’s position is updated at the end of each time step, and the fluid field data and the embryo’s position are recorded every 100 time steps.

### Simulation settings

Our simulations mimic two consecutive processes during ET, embryo injection followed by catheter withdrawal. The simulation settings for both processes are described as follows.

#### Embryo injection

Before the onset of injection, the catheter and the uterine cavity are assumed to be filled with the transferred medium and the uterine fluid, respectively, and both are assumed to be stationary. The uterine fluid is assumed to have similar properties to glycerin with density *ρ*_2_ = 1259.9 kg/m^3^ and viscosity *μ*_2_ = 0.799 Pa·s. The focus of our study is to examine the influence of the viscosity of the transferred medium on the intrauterine fluid flow and the deposition site of embryo during ET. Therefore, the viscosity of the transferred medium is tuned from 0.001 Pa·s (the viscosity of normal saline) to 0.799 Pa·s (the viscosity of uterine fluid). Table 1 lists the viscosities of the transferred medium and the corresponding densities used in our simulations. Specially, the case with *μ*_1_ = 0.001 Pa·s and *ρ*_1_ = 1000 kg/m^3^ is defined as the reference case (Case 1 in Table 1), which represents the ET procedure using normal saline as the transferred medium in clinic. On the other hand, the case with *μ*_1_ = 0.799 Pa·s and *ρ*_1_ = 1259.9 kg/m^3^ is called the equal-viscosity case (Case 7 in Table 1), which mimics the clinical routine of ET using the transferred medium with similar properties to the uterine fluid. The viscosities of other cases are set to be gradually increased between the above mentioned two cases, from Group 1 – low viscosity group to Group 2 – high viscosity group, respectively (Table 1). The injection velocity is assumed to be constant during injection and can be calculated based on the transferred volume and injection time. For the injection time, we simulate the following two procedures respectively: *t*_inj_ = 1 s, which represents a fast injection or single push of the transfer medium (Case 1–7 in Table 1), and *t*_inj_ = 15 s, which represents a slow and gentle injection (Case 8 and 9 in Table 1). The transferred volume is set as 20 μL for all the cases.

**Table 1:** Parameter settings for different cases in the simulations

	Case Index	Viscosity (Pa·s)	Density (kg/m^3^)	Injection time (s)
Reference case	1	0.001	1000	1
Group 1	2	0.005	1001.3	1
	3	0.01	1002.9	
	4	0.05	1016	
Group 2	5	0.1	1032.2	1
	6	0.5	1162.5	
	7	0.799	1259.9	
Slow injection group	8	0.001	1000	15
	9	0.799	1259.9	

#### Catheter withdrawal

The process of catheter withdrawal after injection is simulated for the reference and equal-viscosity cases (Case 1 and 7 in Table 1). The duration of withdrawal is assumed to be 5 s in both cases.

Finally, the parameters of the embryo are set as follows: diameter *d*_*e*_ = 0.1 mm, density *ρ*_*e*_ = 1000 kg/m^3^, initial distance from the tip of catheter *L*_*t*_ = 10 mm and initial velocity ${\left.{\overset{\rightharpoonup }{v}}_e\right|}_{t=0}=0$ for all 10 embryos (*e*_1_–*e*_10_, Fig. 2).

### Focuses of simulations

Our simulation studies focus on the dispersion patterns of the transferred medium and the trajectories of embryo motions during ET. The dispersion pattern of the transferred medium for each case is described by its profile defined as the contour line of *α* = 0.5, i.e. the contour line in which the volume fractions of the transferred medium and uterine fluid are equal. The method for tracking the trajectories of embryos is described in “Prediction of embryo trajectories” section. In order to accurately evaluate the site of embryo deposition, we divided the uterine cavity into two regions. One region between the catheter tip and the uterine fundus is called downstream region, and the other region between the catheter tip and the external ostium is called upstream region (Fig. 1). We also calculate the axial and radial distances of an embryo delivery, which are measured as the axial and radial distances relative to the catheter tip. Additionally, the force of driving the catheter injection is calculated by integrating the pressure over the area of inlet surface.

## Results and Discussion

### Embryo transport during injection

#### Fast injection

**Fig. 3:**
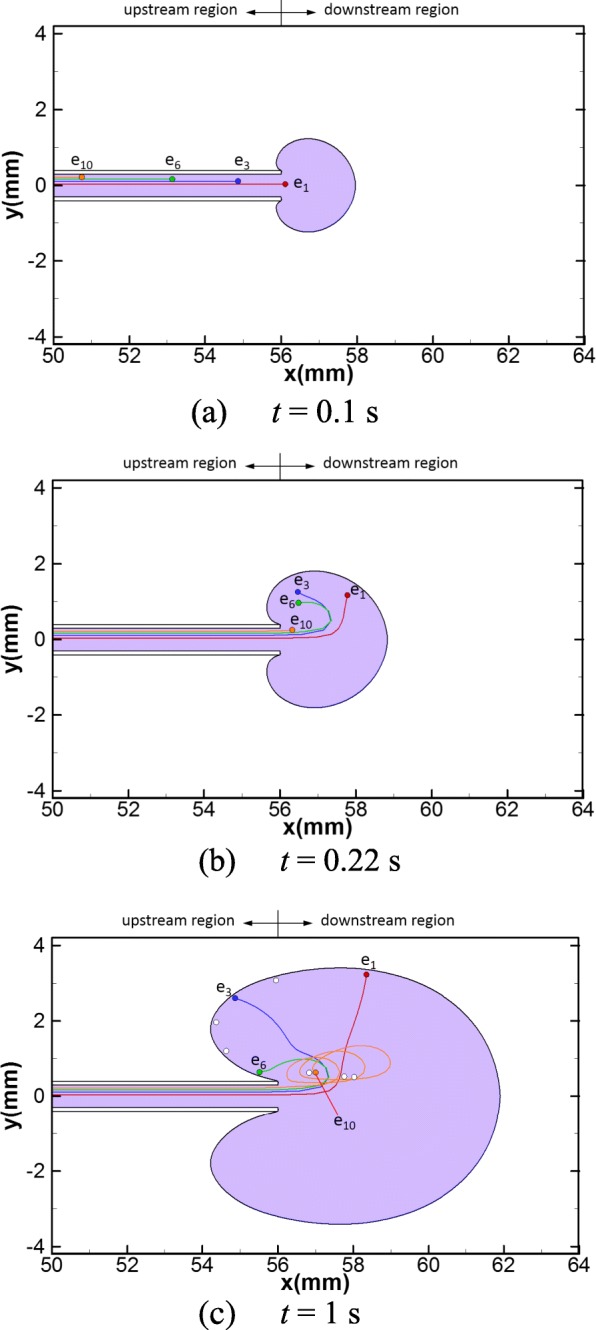
Dispersion pattern of the transferred medium and embryo trajectories for the reference case (Case 1): **a** at *t* = 0.1 s, **b** at *t* = 0.22 s and **c** at *t* = 1 s. The purple area represents the dispersion pattern of the transferred medium. The trajectories of four embryos, *e*_1_, *e*_3_, *e*_6_ and *e*_10_, are depicted. The white dots in panel (**c**) represent the delivery sites of the rest of the 10 embryos

For the reference case (Case 1 in Table 1), the dispersion patterns of the transferred medium and embryo trajectories at three different injection times are shown in Fig. 3a–c, where the trajectories of four embryos (*e*_1_, *e*_3_, *e*_6_ and *e*_10_) are depicted and the final deposition sites of all embryos are marked in Fig. 3c. The shape of the dispersion pattern is similar to an incomplete ellipse and its area increases with the injecting time (Fig. 3a–c, areas colored in purple). From these patterns, we observe two upstream zones spreading towards the cervix near the catheter (Fig. 3a–c). The results also show that the embryos travel along the axis within the catheter before 0.1 s, and their trajectories are straight lines parallel to the axial axis (*x*) (Fig. 3a). After that, they are discharged out of the catheter successively, where the trajectory of *e*_1_ is tilted upwards the radial axis (*y*) and the trajectories of *e*_3_ and *e*_6_ are reversed towards the cervix due to the circulation of vortices formed near the catheter tip (Fig. 3b). At the end of the injection, the embryos with initial locations far from the catheter axis (*e*_7_–*e*_10_) rotate along the vortex and travel towards the fundus, while other embryos (*e*_1_–*e*_6_) move to the outline of the dispersion pattern and five of them (*e*_2_–*e*_6_) fall into the upstream region (Fig. 3c).

**Fig. 4:**
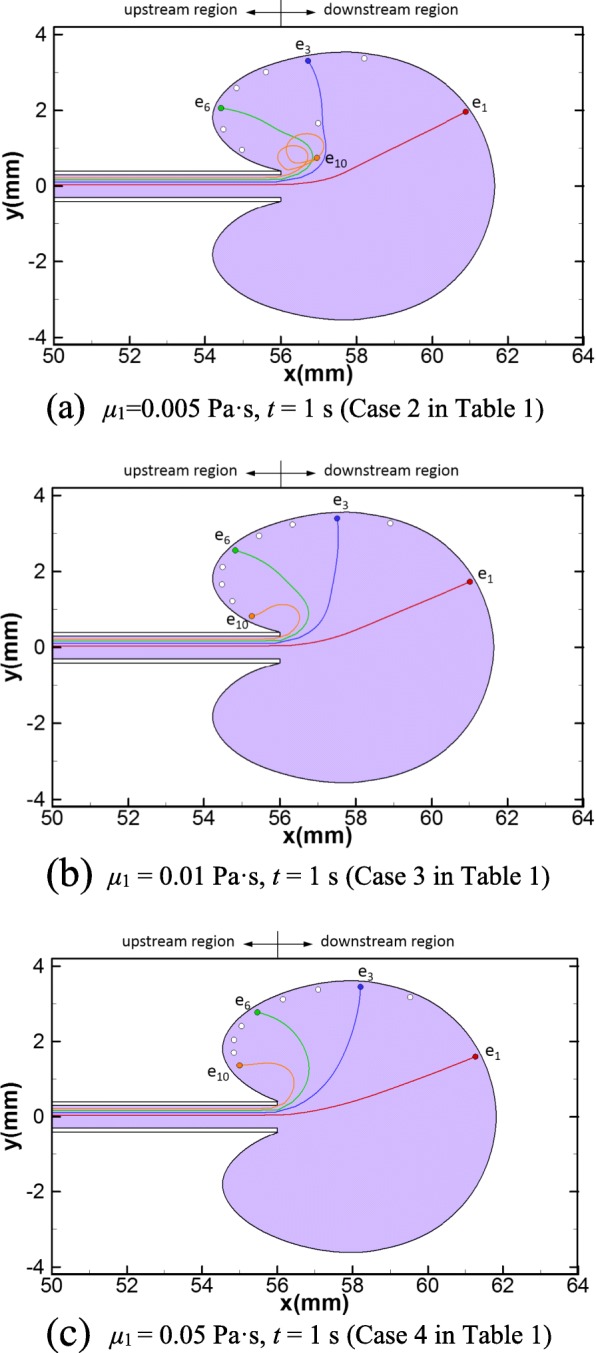
Dispersion pattern of the transferred medium and embryo trajectories for: **a** Case 2 at *t* = 1 s, **b** Case 3 at *t* = 1 s and **c** Case 4 at *t* = 1 s. The purple area represents the dispersion pattern of the transferred medium. The trajectories of four embryos, *e*_1_, *e*_3_, *e*_6_ and *e*_10_, are depicted. The white dots represent the delivery sites of the rest of the 10 embryos

The final dispersion patterns of all cases in Group 1 (Table 1) have similar shape to that of the reference case, which are shown in Fig. 4a–c. When the viscosity of the transferred medium is five times that of the normal saline (Case 2 in Table 1), the embryo trajectories are similar to that of the reference case (Fig. 4a, comparing to Fig. 3c). However, due to the vortices formed close to the catheter tip, two embryos (*e*_9_ and *e*_10_) are transported inside the vortices and they whirl in small areas within the vortices. On the other hand, the embryos initially close to the catheter axis (e.g. *e*_1_) travel further away from the catheter tip and towards the fundus (Fig. 4a, comparing to Fig. 3c). As the viscosity increases (Case 3 and 4 in Table 1), the final delivery sites of all embryos are close to the outline of the dispersion pattern, yet nearly half of them are in the upstream region (Fig. 4b and c).

**Fig. 5:**
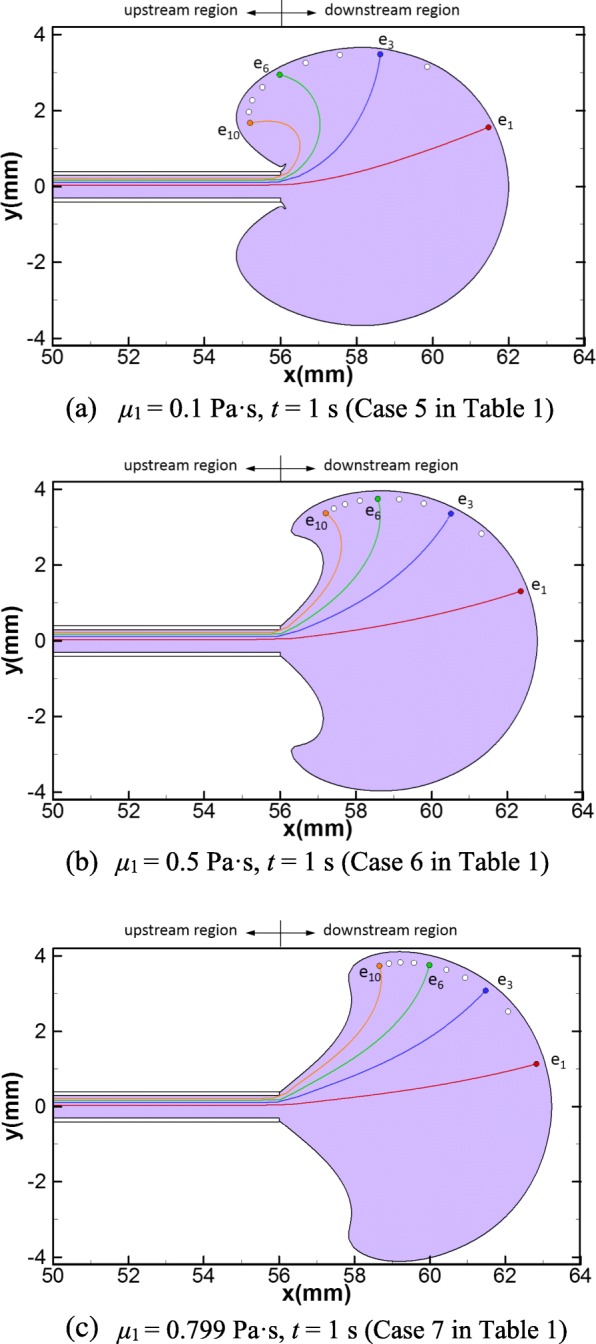
Dispersion pattern of the transferred medium and embryo trajectories for: **a** Case 5 at *t* = 1 s, **b** Case 6 at *t* = 1 s and **c** Case 7 at *t* = 1 s. The purple area represents the dispersion pattern of the transferred medium. The trajectories of four embryos, *e*_1_, *e*_3_, *e*_6_ and *e*_10_, are depicted. The white dots represent the delivery sites of the rest of the 10 embryos

When the viscosity is 100 times that of the normal saline (Case 5 in Table 1), the dispersion pattern is also similar to that of the reference case, yet two cracks on the outline of the dispersion pattern are formed near the catheter tip (Fig. 5a). With the viscosity increased further (Case 6 and 7 in Table 1), these cracks grow larger and reduce the areas of the upstream regions, and the shape of the dispersion pattern becomes similar to a sector (Fig. 5b and c). For the embryo trajectories, no significant difference is observed between Case 4 and 5 (Fig. 4c versus Fig. 5a). However, the trajectories of the cases with high viscosity (*μ*_1_ ≥ 0.5 Pa·s, Case 6 and 7 in Table 1, Fig. 5b and c) are obviously different from the low-viscosity cases (*μ*_1_ < 0.1 Pa·s, Case 1–4 in Table 1, Figs. 3c and 4). For Case 6 and 7, once the embryos are discharged out of the catheter, they tend to move along the radii of the sector and their final locations are at the outline of the sector and in the downstream region (Fig. 5b and c).

#### Slow injection

**Fig. 6:**
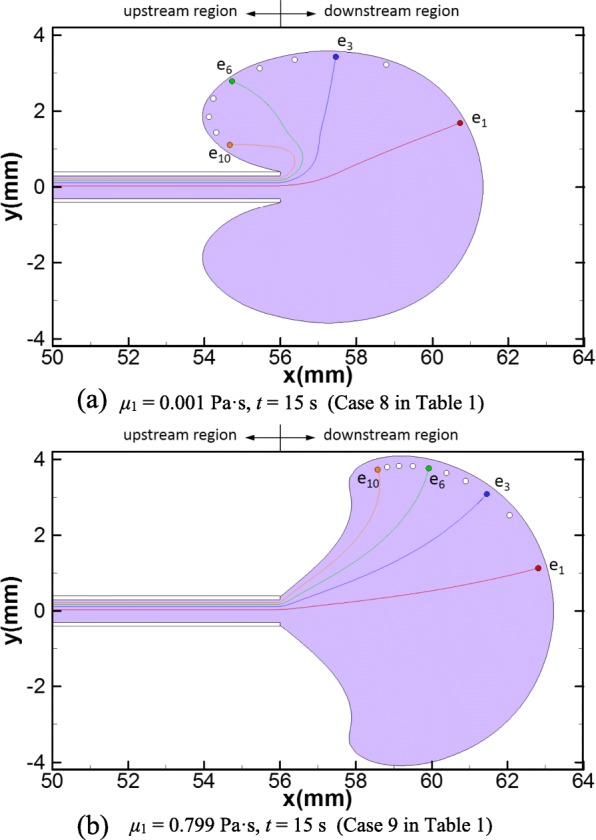
Dispersion pattern of the transferred medium and embryo trajectories with slow injection speed: **a** Case 8 at *t* = 15 s and **b** Case 9 at *t* = 15 s. The purple area represents the dispersion pattern of the transferred medium. The trajectories of four embryos, *e*_1_, *e*_3_, *e*_6_ and *e*_10_, are depicted. The white dots represent the delivery sites of the rest of the 10 embryos

When the injection speed is slow (Case 8 and 9 in Table 1), the dispersion patterns and embryo trajectories (at *t* = 15 s) are shown in Fig. 6. The dispersion pattern for Case 8 (*μ*_1_ = 0.001 Pa·s) has a shape of an incomplete ellipse similar to that of the reference case, yet no embryos travel within the vortices due to the decreased incidence of vortex with slow injection speed. All the embryos are delivered near the outline of the dispersion pattern at the end of injection, and six of them falls in the upstream region (Fig. 6a). For Case 9, the dispersion pattern and embryo trajectories are similar to Case 7 (Fig. 6b, comparing to Fig. 5c).

### Embryo transport during catheter withdrawal

**Fig. 7:**
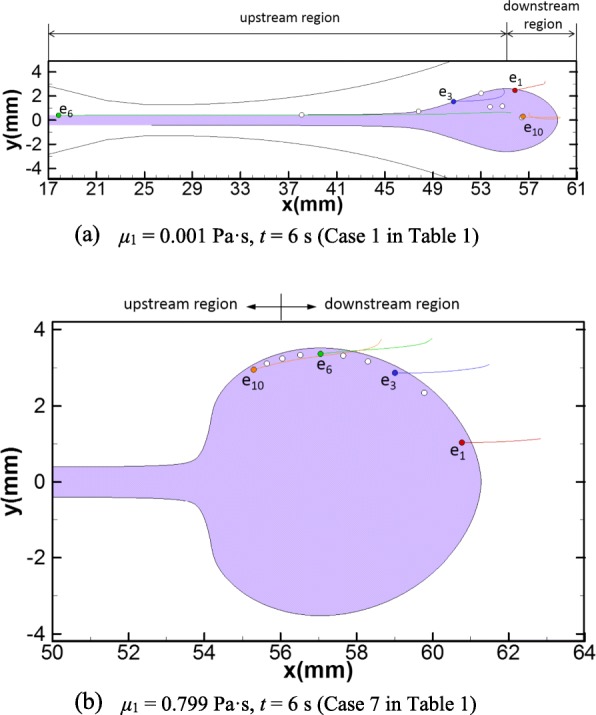
Dispersion pattern of the transferred medium and embryo trajectories after catheter withdrawal for: **a** Case 1 at *t* = 6 s and **b** Case 7 at *t* = 6 s. The purple area represents the dispersion pattern of the transferred medium. The trajectories of four embryos, *e*_1_, *e*_3_, *e*_6_ and *e*_10_, are depicted. The white dots represent the delivery sites of the rest of the 10 embryos

The simulation results of catheter withdrawal after embryo injection are shown in Fig. 7, where the dispersion patterns at the end of catheter withdrawal (*t* = 6 s) and embryo trajectories from the end of injection (*t* = 1 s) to the end of catheter withdrawal (*t* = 6 s) are depicted for the reference case (Case 1, Fig. 7a) and the equal-viscosity case (Case 7, Fig. 7b), respectively. For the reference case, the dispersed region of the transferred medium is elongated by the negative pressure caused by the catheter withdrawal (Fig. 7a). All the embryos are dragged back towards the cervix with an average axial distance of 7.85 mm from their delivered locations at the end of injection. Some embryos delivered into the upstream region at the end of injection (i.e. *e*_3_–*e*_6_) are significantly affected by the catheter withdrawal. Especially, the embryo (i.e. *e*_6_) nearest to the catheter wall at the end of injection is dragged out of the uterine cavity and into the cervix (Fig. 7a). For equal-viscosity case (Case 7), the shape of the dispersion pattern changes from a sector to an incomplete ellipse (Fig. 7b, comparing to Fig. 5c). Though all embryos are also dragged back towards the cervix, their average axial displacement is only 2.80 mm and they all remain within the uterine cavity (Fig. 7b).

### Summary and discussion

One of the major findings in our work is that the viscosities of the transferred medium has significant impacts on the shape of the dispersion pattern of the intrauterine flow. As described above, in the reference case with the same viscosity of normal saline, it shows an incomplete-ellipse-shaped dispersion pattern with two large upstream regions (Fig. 3c), which is consistent with the dispersion pattern found in real experiments by Eytan et al. [12]. As the viscosities of the transferred medium increase, the shape of its dispersion pattern gradually changes from an incomplete ellipse to a sector, and its area in the upstream region progressively reduce and eventually disappear when the viscosity of the transferred medium is close to that of the uterine fluid (Case 2–7, Figs. 4 and 5). These findings are also consistent with the conclusion of Eytan et al. based on real experiments [12].

The viscosity of the transferred medium also crucially affects the motions and the final delivery sites of the embryos. In natural gestations, a high pregnancy rate is achieved if the implantation site of an embryo is close to the fundus, while a high miscarriage rate is expected if the implantation site is in the lower and middle parts of the uterine cavity (the lower and middle parts of the uterine cavity correspond to the upstream region in our study as illustrated in Fig. 1) [18]. Also, unless the openings of the fallopian tubes is dilated usually caused by disease conditions, it is rare that an embryo falls into the fallopian tubes through embryo injection and leads to ectopic pregnancy [18]. Therefore, the favorable delivery site of an embryo during ET should be as close as to the fundus. As discussed in “Embryo transport during injection” section, the trajectories and delivery sites of the embryos for different viscosities of transferred medium are depicted (Figs. 3, 4, 5 and 6). To further quantify the embryo transports, the mean values of the axial and radial transport distances of the 10 embryos at the end of injection for each case are calculated and summarized in Table 2. As the viscosity increases, the mean axial transport distances of the embryos become more and more closer to the fundus (Table 2), which are less than 1 mm for low viscosity cases (Case 1–4, Table 2) but grow to more than 4 mm for the equal-viscosity case (Case 9, Table 2). Also, the mean radial transport distances for all cases are in the range of 1.5 to 3.28 mm, which are far away from the fallopian tube ostium (the usual radial distance between the fallopian tube ostium and the uterine midline is about 16 mm [1]). Therefore, our simulations suggest that using the transferred medium with viscosity close to that of the uterine fluid would significantly enhance the probability of delivering the embryos close to the fundus and thus achieve high successful pregnancy rate.

**Table 2:** Mean embryo transport distances and driven forces of injection for different viscosities and injection speeds

*Case Index*	*Viscosity* (Pa·s)	*Injection speed* (mm/s)	*Axial transport distance* (mm)	*Radial transport distance* (mm)	*Driven force for transferring catheter load* (10^−3^ N)
1	0.001	70.7	0.33	1.50	0.52
2	0.005	70.7	0.41	2.12	1.04
3	0.01	70.7	0.30	2.30	1.68
4	0.05	70.7	0.74	2.50	6.84
5	0.1	70.7	1.13	2.64	13.29
6	0.5	70.7	3.21	3.28	64.89
7	0.799	70.7	4.41	3.27	103.65
8	0.001	4.72	0.09	2.44	0.03
9	0.799	4.72	4.35	3.28	6.90

Another major finding in this work is regarding the optimal condition of the injection speed. Though a high viscosity transferred medium can provide desirable delivery location for the embryo, it may lead to high variations of pressure within the catheter and a high driving force on the embryo during injection, which can injure the embryo [19, 20]. The driving force depends not only on the viscosity of the transferred medium but also the speed of injection. We calculate the driving forces of injection for all cases, which are listed in the last column in Table 2. As expected, the driving force remarkably grows with the increment of the transferred medium viscosity for fast injection cases (Case 1–7, Table 2). For the equal-viscosity case (Case 7, Table 2), the driving force is 200 times that of the reference case (Case 1, Table 2). On the other hand, decreasing the speed of injection can significantly reduce the driving force. For the cases with slow injection speed, the estimated driving forces is 1/15 that of fast injection cases. In addition, no obvious difference is observed for the embryo trajectories and the transport distances comparing the two equal-viscosity cases with fast and slow injection speed (Case 9 versus Case 7 in Table 2. See also Fig. 5c versus Fig. 6b), which indicates that the embryo trajectories of high viscosity transferred medium is not sensitive to injection speed. Based on the above analysis, we recommend that using an equal-viscosity transferred medium with a slow injection speed for ET procedures in clinic.

Last but not least, our work shows that the delivery of embryos during ET also depends on catheter withdrawal, the successive procedure of embryo injection. According to the simulation results, all embryos are dragged back towards the cervix from their injected positions caused by catheter withdrawal (Fig. 7). For the reference case, one embryo is even dragged into the cervix (*e*_6_ in Fig. 7a). This observation is consistent with a clinical study that reported 8.7% patients undergoing IVF have embryos in the cervix or on the speculum after routine ET [21]. For the low viscosity cases (*μ*_1_ < 0.1 Pa·s), nearly half of the embryos tracked in the simulations are delivered into the upstream regions (Case 1–4, Figs. 3c and 4a–c). It is reasonable to conjecture that the catheter withdrawal may drag embryos into the middle or lower parts of the uterine cavity and even into the cervix, which may lead to the failure of IVF. However, increasing the viscosity of the transferred medium close to that of the uterine fluid results in much smaller dragging distances towards the cervix for the embryos (Fig. 7b, comparing with Fig. 7a). Therefore, increasing the viscosity of the transfer medium can decrease the chance that the embryo is dragged out of the uterine cavity during catheter withdrawal, which is in agreement with one reported clinical research [22].

## Conclusions

In summary, a computational model is developed to simulate the intrauterine mixing flow and embryo trajectories during embryo injection and catheter withdrawal of ET. The simulation results show that the dispersion pattern of the transferred medium and the final delivery sites of the embryos are significantly influenced by the viscosity of the transferred medium. Specifically, increasing the transferred medium viscosity close to that of the uterine fluid can enhance the probability of the embryos arriving at the fundus and keep them from being dragged backward to the cervix by catheter withdrawal. Based on our work, the protocol that uses a transferred medium with similar viscosity to that of the uterine fluid and a slow injection speed is the optimal condition, and thus should be recommended for real ET procedures in clinic.

## Abbreviations

ET: Embryo transfer
IVF: In vitro fertilization
s: seconds
